# Biosilicated collagen/β-tricalcium phosphate composites as a BMP-2-delivering bone‐graft substitute for accelerated craniofacial bone regeneration

**DOI:** 10.1186/s40824-021-00214-w

**Published:** 2021-04-21

**Authors:** Dong Keon Lee, Mi-Ran Ki, Euy Hyun Kim, Chang-Joo Park, Jae Jun Ryu, Hyon Seok Jang, Seung Pil Pack, Yun Kee Jo, Sang Ho Jun

**Affiliations:** 1 Department of Oral and Maxillofacial Surgery, Korea University Anam Hospital, 02841 Seoul, Korea; 2 Department of Biotechnology and Bioinformatics, Korea University, 30019 Sejong, Korea; 3 Division of Oral and Maxillofacial Surgery, Department of Dentistry, College of Medicine, Hanyang University, 04763 Seoul, Korea; 4 Department of Prosthodontics, Korea University Anam Hospital, 02841 Seoul, Korea; 5 Department of Oral and Maxillofacial Surgery, Korea University Ansan Hospital, 15355 Ansan, Korea; 6 Department of Biomedical Convergence Science and Technology, School of Convergence, Kyungpook National University, 41566 Daegu, Korea; 7 Cell and Matrix Research Institute, Kyungpook National University, 41566 Daegu, Korea

**Keywords:** Osteoinductive bone substitute, Collagen/β-TCP composite, Biosilicification, Bone morphogenetic protein-2 (BMP-2), Biomimetic materials

## Abstract

**Background:**

Bioceramic β-tricalcium phosphate (β-TCP) is used as a bone-grafting material and a therapeutic drug carrier for treatment of bone defects in the oral and maxillofacial regions due to the osteoconductivity and biocompatibility. However, the low mechanical strength and limited osteoinductivity of β-TCP agglomerate restrict bone regenerating performance in clinical settings.

**Methods:**

Herein, a biomimetic composite is proposed as a bone morphogenetic protein-2 (BMP-2)-delivering bone graft substitute to achieve a robust bone grafting and augmented bone regeneration.

**Results:**

The sequential processes of brown algae-inspired biosilicification and collagen coating on the surface of β-TCP enable the effective incorporation of BMP-2 into the coating layer without losing its bioactivity. The sustained delivery of BMP-2 from the biosilicated collagen and β-TCP composites promoted *in vitro* osteogenic behaviors of pre-osteoblasts and remarkedly accelerated *in vivo* bone regeneration within a rat calvarial bone defect.

**Conclusions:**

Our multicomposite bone substitutes can be practically applied to improve bone tissue growth in bone grafting applications with further expansion to general bone tissue engineering.

## Background

Bone grafting materials have been extensively used for the treatment of oral and maxillofacial bone defects arising from trauma, periodontal disease, surgical bone resection and congenital defects [1]. Autogenous bone has been considered as a gold standard bone graft because of its outstanding osteogenic, osteoconductive, and osteoinductive properties; but limited availability, invasive harvesting procedure and high donor-site morbidity are major hurdles for practical applications of autogenous bone grafting [2, 3]. As an alternative to autogenous bone, β-tricalcium phosphate (β-TCP) has attracted in bone grafting due to excellent osteoconductivity and resorbability [4, 5]. The composition and microstructure of this calcium phosphate ceramic are very close to those of natural bone, which is advantageous to provide a desirable environment for bone tissue regeneration [6, 7]. However, the use of β-TCP often gives rise to compromised bone regeneration in regions where the osseous environment is inactive owing to its limited osteoinductivity [8, 9]. In addition, an agglomerate of β-TCP is susceptible to catastrophic failure due to its inferior fracture toughness and high Young’s modulus, which restricts its clinical use on load-bearing sites [10].

Bone morphogenetic protein-2 (BMP-2), an osteoinductive protein that strongly induces the recruitment and differentiation of mesenchymal progenitor cells into mature osteoblasts, thus generating a bony matrix, has been widely utilized to treat bone under approval of the United States Food and Drug Administration (FDA) [11, 12]. Several types of β-TCP carriers have been developed for delivery of BMP-2 to actively induce augmented bone formation, while providing an osteoconductive core for promoting ingrowth of an osteogenic responses within the bone defect [13]. However, initial burst release pattern obtained with the high resorption rate of β-TCP may lead to reduced bone-inducing activity and potential adverse effects in clinical settings [14, 15].

In the present work, we propose a hybrid type of BMP-2-delivering bone-graft substitute by employing two biomimetic strategies. First, natural bone is composed of organic-inorganic composites where calcium phosphate nanocrystals are precipitated onto collagen fibers [16]. Collagen is a major component of the bone extracellular matrix (ECM) which has excellent biocompatibility and biodegradability as well as bioactivity for facilitating cell adhesion [16, 17]. Considering that the mechanical property of hybrid composite strongly depends on interfacial bridging between organic polymer and inorganic particles [18], the incorporation of β-TCP with collagen (Col/β-TCP) could be an attractive approach to accomplish tailored delivery of BMP-2 and essential mechanical stiffness at different stage of bone regeneration. Second, the skeletal architectures of brown algae are composed of hierarchical silica nanostructures containing organic components that rapidly form silica nanoparticles (SiNPs) under physiological conditions [19, 20]. This natural biosilica formation for protection and mechanical support has provided a captivating source as a more attractive approach for silica synthesis in biomedical applications over the conventional sol-gel methods due to mild processing in ambient temperature and pH without the presence of hazardous chemicals. In particular, biosilica has been diversely utilized as a biocompatible, osteoinductive inorganic material for bone tissue engineering as well as a drug vehicle for sustained release of therapeutic molecules due to the rich hydroxyl groups of its hydrophilic surface [21]. Thus, we hypothesized that biosilicification of the Col/β-TCP composite confers enhanced bone-forming activity and sufficient structural stability, while allowing a robust adsorption of positively charged BMP-2 onto the negatively charged biosilica [22], eventually making it suitable drug-releasing bone-graft substitute for load-bearing bone defects.

Here, we prepared biosilicated Col/β-TCP composites (Col/Si-β-TCP) using a silica-forming peptide derived from a brown algae *Ectocarpus siliculusus* and assessed potential applicability as a BMP-2-delivering bone-graft substitute for enhancing *in vitro* osteogenic behaviors and accelerating *in vivo* bone growth.

## Methods

### Preparation of BMP-2-loaded Col/Si-β-TCP composite block

**Fig. 1:**
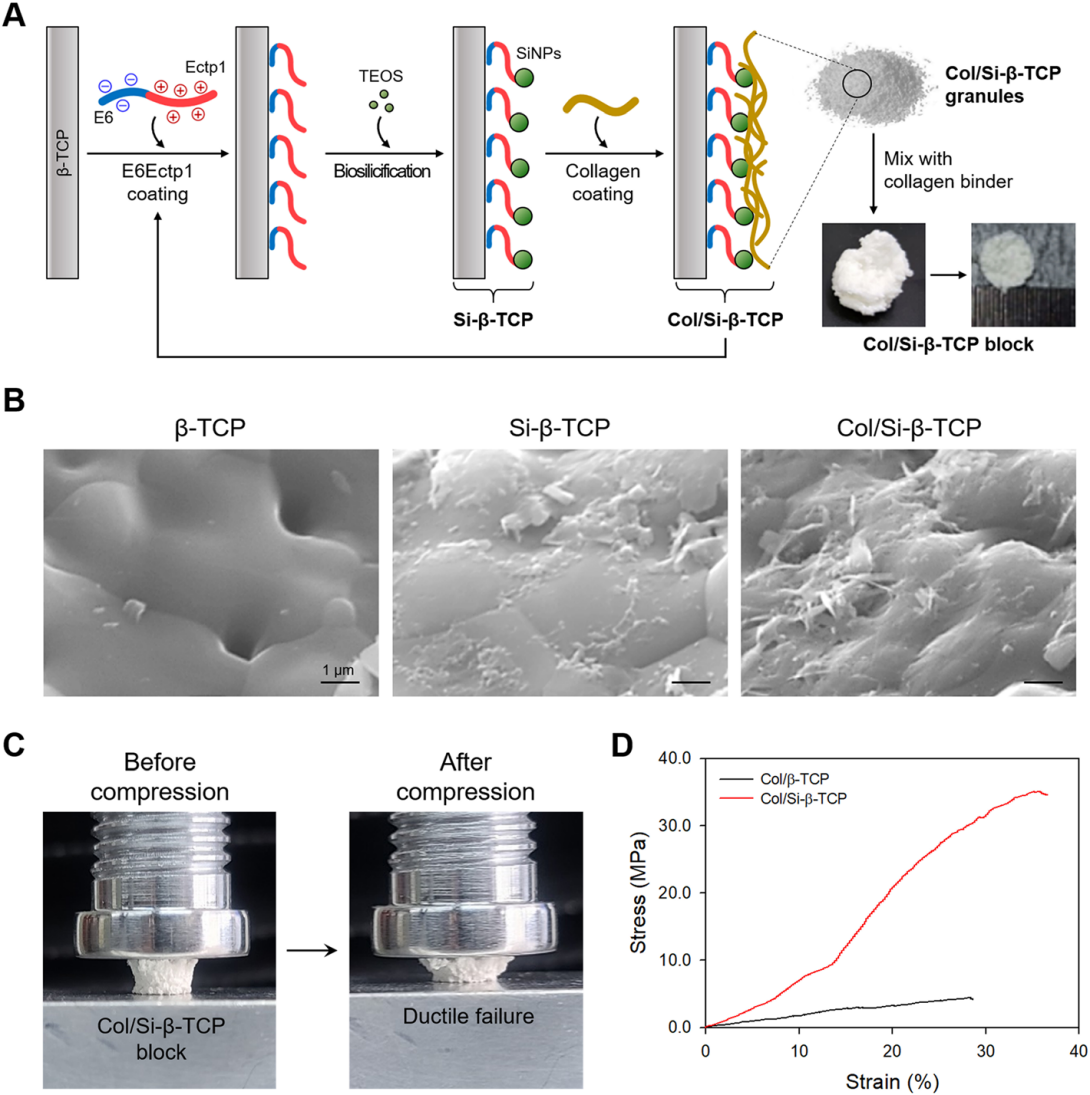
Preparation and characterization of the Col/Si-β-TCP composite block. **a** Schematic illustration of preparation of the Col/Si-β-TCP composites by the sequential process of E6Ectp1 coating, silicification and collagen coating and the Col/Si-β-TCP block by mixing with collagen binder. **b** SEM images of the non-treated β-TCP, the biosilicated β-TCP (Si-β-TCP) and the collagen-coated Si-β-TCP (Col/Si-β-TCP). Scale bar is 1 μm. **c** Disk-shaped bone blocks were loaded in uniaxial compression. The bone blocks showed ductile failure under the applied loading. **d** Strain-stress curve of the Col/β-TCP and the Col/Si-β-TCP bone block

For the coating of E6Ectp1 (EEEEEEGGSSRSSSHRRHDHHDHRRGS) peptide (Genscript, Piscataway, NJ, USA), one gram of porous β-TCP particles (diameter = 0.6-1 mm; average pore size = 300 μm; porosity = 70–80 %; CG Bio, Seoul, Korea) was added in 1 mL of the E6Ectp1 peptide (0.1–0.5 mg mL^− 1^) at 4 °C for overnight, followed by a gentle rinsing to remove unbound peptides [23]. For biosilicification, the silica forming peptide (SFP)-bound β-TCP particles were immersed in a mixture of 1.5 % (v/v) silicic acid (Sigma), 36 mM choline chloride (Sigma) and 46 % (v/v) ethanol [24] for different incubation periods (6–24 h) followed by washing with distilled water (DW) 10 times for removal of residual silicic acid. Then, the biosilicated β-TCP (Si-β-TCP) and 0.05 % (v/v) porcine collagen (FlexiCol®; Sigma, St. Louis, MO, USA) in phosphate buffered saline (PBS; Sigma), were mixed with a gentle agitation for 6 h. After washing with DW once, the resulting Col/Si-β-TCP composites were placed in one more repeated process of SFP coating, silicification and collagen coating (Fig. 1 a). The prepared Col/Si-β-TCP composite particles were dried. All procedures were carried out under sterile condition. The morphology of the Col/Si-β-TCP composites was analysed by field emission-scanning electron microscope (FE-SEM; JSM-6700 F; JEOL, Tokyo, Japan).

To prepare disk-type blocks, 3.6 g of the Col/Si-β-TCP or non-treated β-TCP particles were homogeneously mixed with 0.4 g of bovine type I collagen dough (CG Bio) in 0.2 M acetic acid, which corresponds to 10 % (w/w) of the total mass of bone graft particles. The mixture of collagen binder and bone graft particles was placed in a disk-shaped silicone mold with a diameter of 6 mm and a height of 2 mm. The resulting blocks were lyophilized and dialyzed in a sterile PBS for 24 h, followed by another dialysis in DW for 24 h. They were dried at room temperature and then sterilized by gamma irradiation of 15 kGy for further use. For loading of BMP-2, one µg of recombinant human BMP-2 (rhBMP-2; CG Bio) was adsorbed to ~ 65 mg of Col/Si-β-TCP block or β-TCP block at room temperature for 30 min, and then washed once with PBS to remove unbound rhBMP-2. The concentration of BMP-2 was quantified with human BMP-2 ELISA kit (Sino Biological, Wayne, PA, USA) according to the manufacturer’s instructions. The BMP-2 entrapment efficiency was calculated using the following formula:

$$Entrapmentefficiency\left(EE\%\right)=\frac{(TotalamountofaddedBMP-2)--(AmountoffreeBMP-2)}{(TotalamountofaddedBMP-2)}\times100$$

### Evaluation of mechanical strength

The compressive behavior of the prepared bone graft blocks was evaluated *via* an unconfined compression test. The specimens of Col/Si-β-TCP block or the Col/β-TCP block in a diameter of 6 mm and a height of 5 mm were placed on the center of loading platens in a universal testing machine instrument (Taewon Tech, Bucheon, Korea) and compressed to failure at a rate of 1 mm s^-1^.

### Evaluation of cell viability

Mouse pre-osteoblast MC3T3-E1 subclone 4 cells (ATCC® CRL-2593™; ATCC, Manassas, VA, USA) were cultured and maintained in minimal essential medium-α (MEM-α; HyClone, Logan, UT, USA) supplemented with 10 % (v/v) fetal bovine serum (FBS; Lonza, Verviers, Belgium) and 1 % (v/v) penicillin/streptomycin (HyClone) at 37 °C in a humidified atmosphere with 5 % CO_2_.

MC3T3-E1 cells were seeded onto the bone graft blocks at a density of 2.5 × 10^4^ cells/well. Before each assay, the bone blocks were sterilized under UV radiation and then soaked in growth media overnight. A disk-shaped collagen plug (TeruPlug®; Olympus Termo Biomaterials, Tokyo, Japan) was used as a negative control. The culture medium was replaced every 3 day and cell proliferation level on each bone block was investigated at predetermined time points by 3-(4,5-dimethylthiazol-2-yl)-5-(3-carboxymethoxyphenyl)-2-(4-sulfophenyl)-2 H-tetrazolium (MTS) assay (Promega), according to the manufacturer’s instructions. The absorbance at 490 nm was measured using an UV-vis spectrophotometer (Infinite M200 PRO NanoQuant; TECAN, Männedorf, Switzerland). The cell numbers were normalized to that of the bare collagen plug at Day 1.

### Evaluation of osteogenic differentiation

To identify the osteoinductivity of the BMP-2-loaded Col/Si-β-TCP block, MC3T3-E1 cells after ~ 90 % confluence were incubated in a culture medium supplemented with 50 µg mL^− 1^ ascorbic acid (Sigma) and 10 mM β-glycerophosphate (Sigma) and the medium was changed every 3 days.

For phenotypic analysis of osteogenic differentiation, alkaline phosphatase (ALP) activity was determined at 3, 7 and 14 days after the induction of differentiation using a *p*-nitrophenyl phosphate (*p*NPP)-based ALP assay kit (Sigma) according to the manufacturer’s instructions. The values of ALP activity were divided by the cell numbers and normalized to that on the bare collagen plug at Day 3.

To evaluate the expression of osteogenic genes including bone sialoprotein (BSP) and osteocalcin (OCN), total cellular RNAs were extracted from cultured cells at 3, 10 and 14 days after the induction of differentiation using a TRIzol® reagent (Thermo Fisher Scientific, Waltham, MA, USA). Then, cDNA synthesis was performed by the reverse transcription of purified RNA using ReverTra Ace® RT Master Mix (Toyobo, Japan). The cDNAs were amplified by real-time reverse transcription-quantitative polymerase chain reaction (RT-qPCR) with the oligonucleotide primers for the selected genes (Table 1) using a StepOnePlus real-time PCR system (Thermo Fisher Scientific). The amounts of target genes were expressed as the mean fold over the expression level of cells on the bare collagen plug. The housekeeping gene glyceraldehyde 3-phosphate dehydrogenase (GAPDH) was used as the reference gene.

**Table 1:** Primers used for qRT-PCR in this study

Marker gene	Primer sequence (5’ → 3’)		T_m_ (°C)	Reference
COL	Forward	CTTCACCTACAGCACCCTTGTG	59	This study
	Reverse	TGACTGTCTTGCCCCAAGTTC	58	This study
OCN	Forward	GTGAGCTTAACCCTGCTTGTGA	58	This study
	Reverse	TGCGTTTGTAGGCGGTCTTC	60	This study
GAPDH	Forward	CCTGGCCAAGGTCATCCATG	62	Ref. [20]
	Reverse	GCAGGAGACAACCTGGTCCT	62	Ref. [20]

### Animal surgery

Twelve male Sprague-Dawley rats (OrientBio, Seongnam, Korea) weighing ~ 250-300 g were quarantined two per cage and pre-housed in a specific-pathogen-free level-2 (SPF-2) facility for 2 weeks. The rats were allowed free access to take water and food in a temperature- and humidity-controlled room (23 °C, 50 %) with a 12/12 h day/night cycle. Animals were randomly divided and all surgical procedures were performed in a strictly sterile environment. The animals were reared and all procedures were conducted in a facility accredited by the institutional animal care and use committee of Korea University.

Each rat was anesthetized by an intramuscular injection of 10 mg kg^− 1^ Rompun (xylazine; Bayer, Leverkusen, Germany) and 80 mg kg^− 1^ Ketamine (Yuhan, Seoul, Korea). After sterilization with betadine, 2 % lidocaine containing epinephrine (1: 100,000) was injected into the epidermis of surgical site. The scalp was incised carefully and each exposed calvarium was punctured to form a critical-size defect using an 8 mm-diameter trephine bur under sterile saline irrigation. The defect was filled with each bone graft block (the bare collagen plug, the Col/β-TCP block, the Col/β-TCP@BMP-2 block and the Col/Si-β-TCP@BMP-2 block), and the incised periosteum and skin were sealed in layers with 4 − 0 Vicryl® absorbable sutures (Ethicon, Somerville, NJ, USA).

### Radiographic and histological analyses

Each rat was anesthetized as in the surgery and radiographic images of the calvarial specimens were obtained at predetermined time points using X-ray microcomputed tomography (micro-CT; SkyScan 1176; Bruker-microCT, Aarselaar, Belgium) to obtain sequential data over the healing periods. Two-dimensional (2D) images (12.78 μm per slice) were obtained with a 0.5 mm aluminum filter, 60 kV tube voltage and 417 µA tube current, and reconstructed using a reconstruction software (NRecon v.1.4.4; Bruker-microCT). The volume fraction of newly formed bone (BV/TV) was determined from the binary images with a circular region of interest (ROI; 8 mm diameter and 1 mm height) using an image analysis program (CTAn v.1.6.0; Bruker-microCT) in a gray scale range of 60–180.

The calvarial specimens were fixed with 10 % (v/v) formalin (Sigma) for 24 h after euthanization at 8 weeks post-surgery. The specimens were decalcified in a decal solution (Calci-Clear Rapid; National Diagnostics, Somerville, NJ, USA) for 24 h and subsequently embedded in paraffin. After the deparaffinization and hydration processes, 3 μm-thick tissue sections were stained with Masson’s trichrome (MT; Sigma) and hematoxylin-eosin (H&E; Sigma). Histology photographs were obtained using a BX-41 microscope (Olympus) and analyzed using an image analysis program (CaseViewer v.2.0; 3DHISTECH, Budapest, Hungary).

### Statistical analysis

*In vitro* data were obtained from at least three independent experiments, and three samples were measured for each experiment. *In vivo* experiments were performed using four individual rats for each group (*n* = 4). The significance of the data obtained from each group was statistically analysed by Student’s t-test (for a two-group comparison) or one-way analysis of variance (ANOVA) test with Turkey’s HSD post-hoc test (for a multiple comparison). All data represent the mean ± standard deviation with statistical significance. All statistical analyses were performed using SPSS software (v. 24.0; IBM, Armonk, NY, USA).

## Results

### Fabrication and characterization of the Col/Si-β-TCP composite block

We fabricated the Col/Si-β-TCP particles by repeating the sequential process of E6Ectp1 peptide coating, silicification and collagen coating twice (Fig. 1 a). The silica-forming Ectp1 peptide has been known to be capable of binding to mineral surface as well as allowing rapid silica deposition from silicic acid [20]. The sequence of negatively charged hexaglutamate (E6) was fused into the N-terminus of Ectp1 peptide, enabling the physical adsorption onto the surface of β-TCP particles *via* ionic interactions with the underlying positively charged calcium ions. Silica deposition on the E6Ectp1-coated β-TCP particle led to rougher surface morphology compared to that of the non-treated β-TCP as determined by SEM, indicating an effective immobilization of biosilica onto the underlying surface of β-TCP (Fig. 1b). In addition, porcine skin-derived collagen was coated onto the Si-β-TCP particles to achieve the modulated loading and release of BMP-2. The Col/Si-β-TCP and β-TCP particles were bound in a disk-shaped block using a collagen dough as a binder. The resulting Col/Si-β-TCP block exhibited a higher entrapment of BMP-2 (~ 91 %; 14 ng BMP-2 per mg of block) compared to the Col/β-TCP block (~ 77 %; 12 ng BMP-2 per mg of block). From the uniaxial compression results, both the Col/β-TCP and Col/Si-β-TCP blocks were slowly distorted and showed ductile failures (Fig. 1 c). The Col/Si-β-TCP block exhibited a 10-fold higher compressive strength (~ 41 MPa) compared to the Col/β-TCP block (~ 4 MPa) (Fig. 1d).

### ***In vitro*** osteogenic cell behaviors on the BMP-2-loaded Col/Si-β-TCP block

**Fig. 2:**
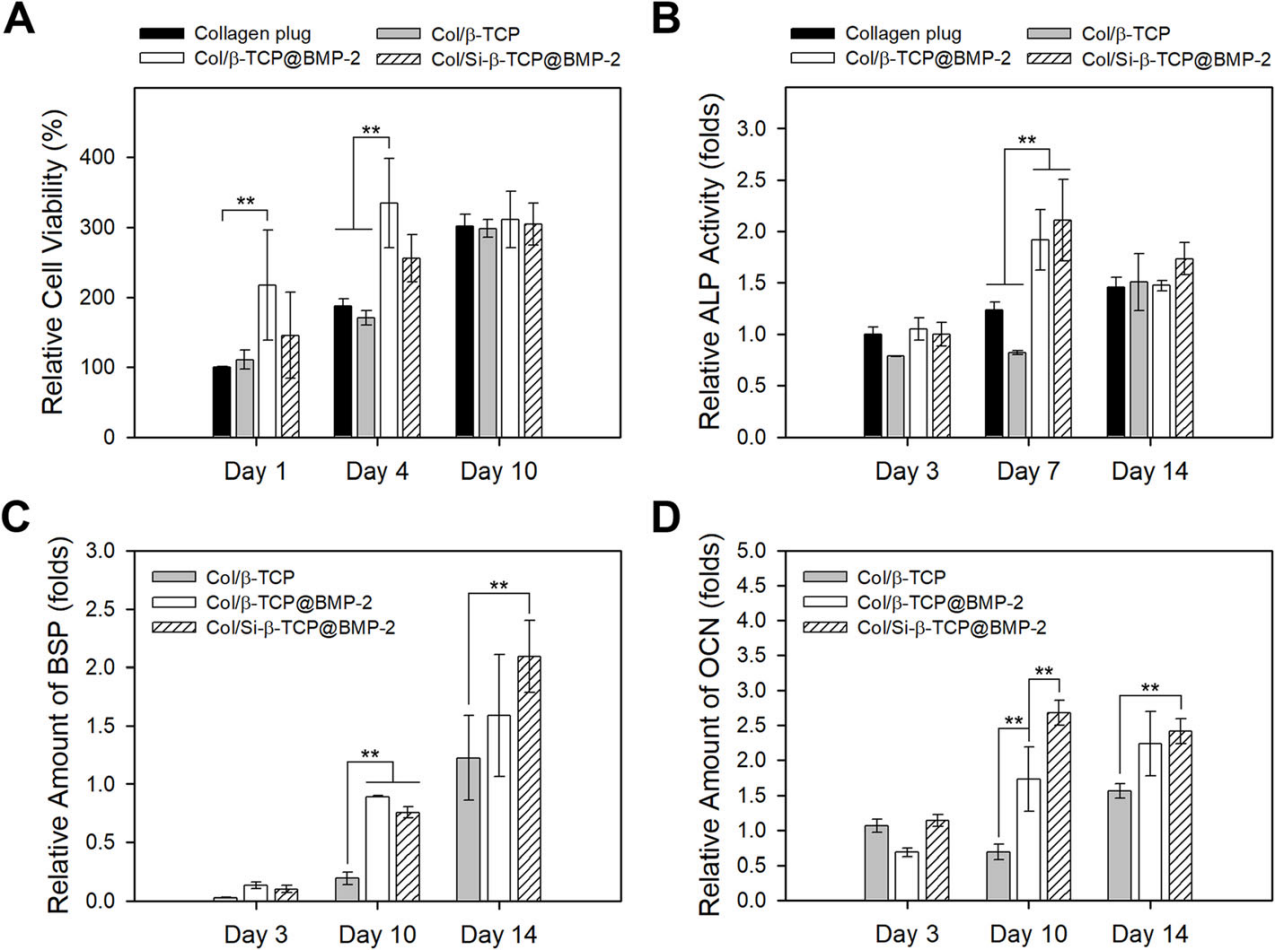
*In vitro* osteogenic behaviors of pre-osteoblasts on the bone-graft surfaces. **a** Cell counts at 1, 4 and 10 days post-seeding measured by MTS assay. **b** ALP activity at 3, 7 and 14 days after induction of differentiation. The relative ALP activity values per each cell were obtained by normalizing to the initial value (for Day 3) of the collagen plug (negative control). The expressions of (**c**) BSP and (**d**) OCN at 3, 10 and 14 days after induction of differentiation. The relative gene expression levels were obtained as the mean fold over the value for the collagen plug (negative control) at the corresponding time points. The data represent the mean ± standard deviation with statistical significance (**p < 0.01)

To identify the biochemical features of Col/Si-β-TCP composite as a BMP-2-delivering bone-graft substitute for regulating the essential cellular functions during bone formation, the osteogenic cell behaviors of pre-osteoblasts were analysed *in vitro*. A bare collagen plug and a BMP-2-free Col/β-TCP block were employed as control groups. Given that ideal bone substitutes should not cause cytotoxicity, we measured the cell viability of pre-osteoblasts on each sample block at 1, 4 and 10 days after seeding. Cells on all sample blocks exhibited more than 90 % viability over the incubation period and a normal growth as the culture time increased, indicating a negligible potential cytotoxicity of the blocks (Fig. 2 a).

One of phenotypic markers related to osteoblast differentiation is ALP, an important glycoprotein for inorganic pyrophosphate metabolism in the maturation phase of osteogenic differentiation [25, 26]. From the colorimetric assay, the BMP-2-loaded Col/β-TCP (Col/β-TCP@BMP-2) block and the BMP-2-loaded Col/Si-β-TCP block (Col/Si-β-TCP@BMP-2) block showed a markedly higher level of ALP activity at 7 days of culture than the bare collagen plug and the BMP-2-free Col/β-TCP block (Fig. 2b). The increase in ALP activity on the Col/Si-β-TCP@BMP-2 block remained even after 14 days of culture, suggesting a sustained delivery of BMP-2 from the Col-Si-β-TCP block.

To verify the osteoblastic commitments of pre-osteoblasts on the bone grafting blocks, the expression levels of osteoblast-associated marker genes including BSP and OCN were measured using real-time qRT-PCR. BSP is a main protein in bone matrix for apatite nucleation during the early mineralization phase, and OCN is primarily expressed in the final stage of matrix mineralization of mature osteoblasts and absorbs hydroxyapatite minerals [25, 26]. We found highly enhanced expression levels of BSP and OCN on the BMP-2-loaded bone graft blocks at 10 and 14 days of culture, which indicates an osteoinductive effect of BMP-2 released from the blocks (Fig. 2 c,d). In particular, the Col-Si-β-TCP@BMP-2 block exhibited the highest upregulation of OCN at 10 and 14 days. Considering the general characteristic of OCN to be expressed in the late stage of osteogenic differentiation, these results can be attributed to the prolonged retention of BMP-2 within the Col/Si-β-TCP block and its sustained release after a certain period of incubation.

### ***In vivo*** bone regeneration on the BMP-2-loaded Col/Si-β-TCP block

**Fig. 3:**
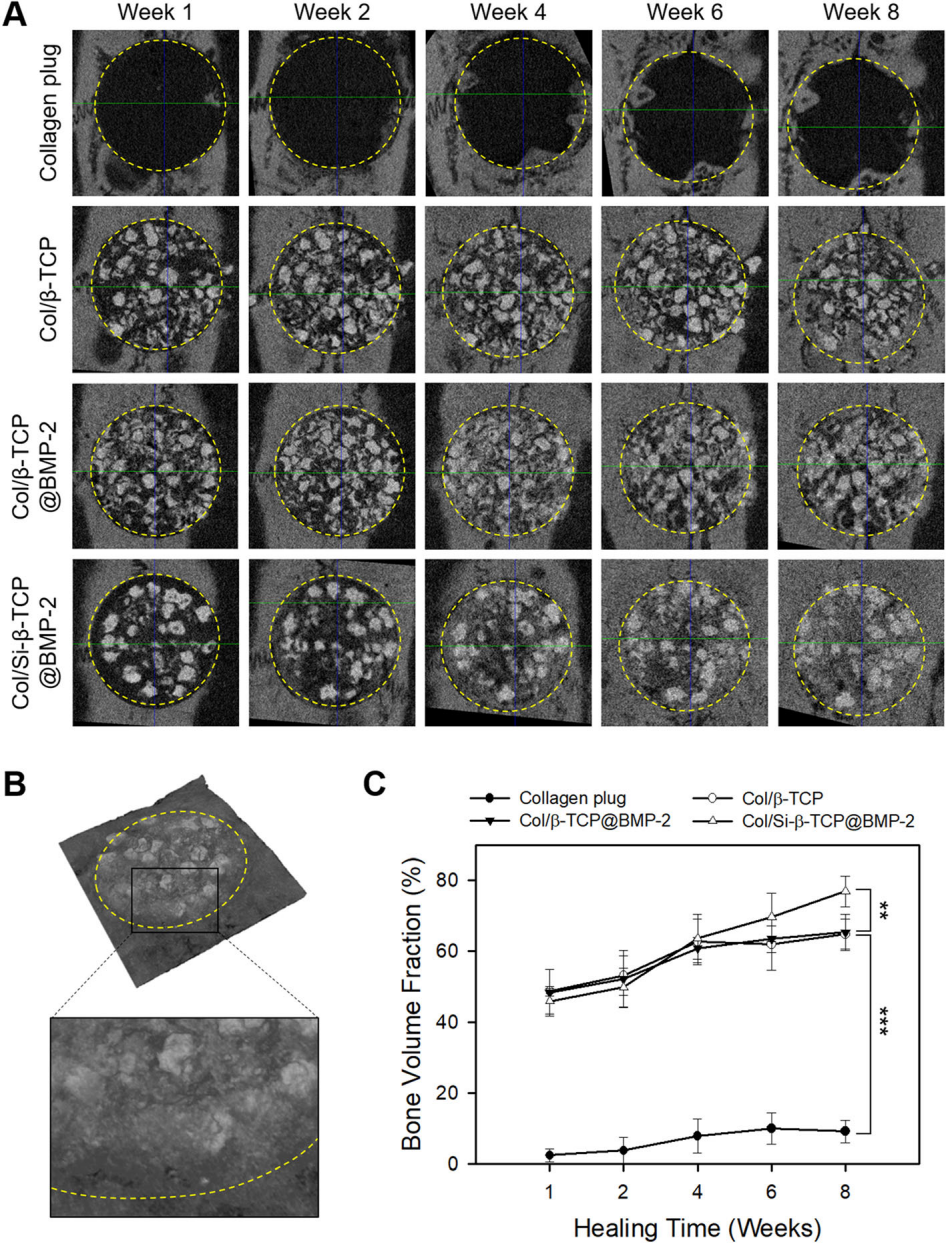
Micro-CT analyses on bone regeneration in a rat calvarial defect. Images for (**a**) a transverse view of all experimental groups over the healing period of 8 weeks and (**b**) a 3d rendering of the Col/Si-β-TCP group at 8 weeks post-surgery. The yellow dotted line indicates the initial marginal boundary of a bone defect. **c** Bone volume fraction of the defect site. The data represent the mean ± standard deviation with statistical significance (**p < 0.01; ***p < 0.005)

Each sample bone block was implanted into crucial-sized rat calvarial defect sites to identify *in vivo* ability for stimulating bone regeneration. The formation of new bone at the defect site was sequentially monitored using a live animal X-ray micro-CT imaging system at 1, 2, 4 and 8 weeks after surgical implantation (Fig. 3 a). In the 3D reconstructed images, all the β-TCP bone grafting groups, regardless of the loading of BMP-2, demonstrated a significant recovery of defects by newly formed bones from the periphery to the center without any detectable loss of β-TCP particles from the defect sites. The Col/Si-β-TCP@BMP-2 group showed the fastest bone healing rate to recover the defect after week 4 and almost fully integrated pattern of bone graft particles with newly formed bone after week 6. In addition, the defect of the Col/Si-β-TCP@BMP-2 group was packed with a remarkably compact and radiopaque microstructure at the end of healing period (Fig. 3b). Correspondingly, at week 8, the bone volume fraction within the defect of the Col/Si-β-TCP@BMP-2 group (~ 77 %) was significantly higher than that of the Col/β-TCP@BMP-2 group (~ 65 %) and the Col/β-TCP group (~ 65 %) (Fig. 3 c). The bare collagen plug group failed to bridge the bone defect with less recovery (~ 9 %).

**Fig. 4:**
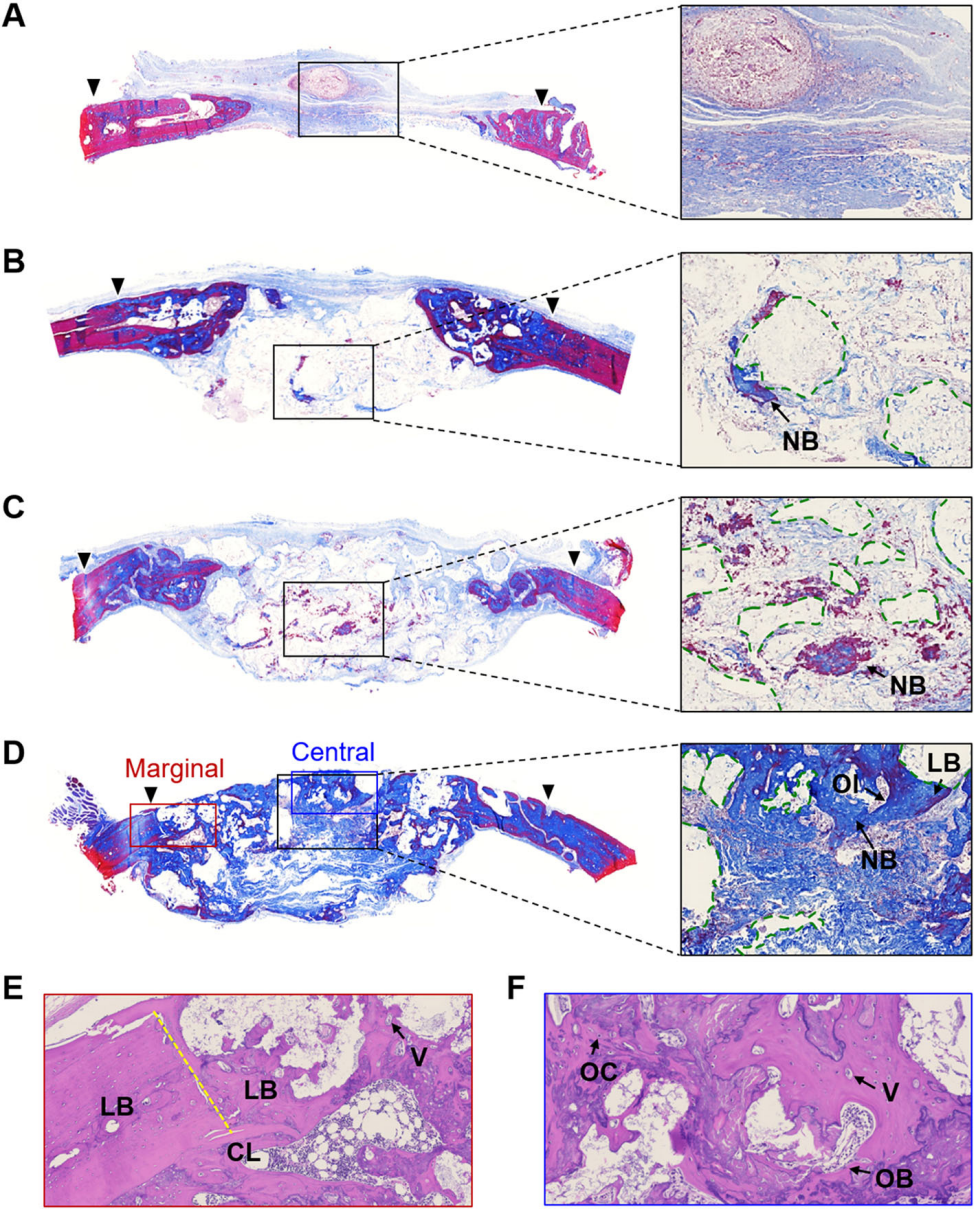
Histological analyses of bone regeneration in a rat calvarial defect at 8 weeks post-surgery. Images of MT-stained sections for (**a**) the bare collagen group, (**b**) the Col/β-TCP group, **c** the Col/β-TCP@BMP-2 group and (**d**) the Col/Si-β-TCP@BMP-2 group. The black triangles indicate the marginal defect point, the black boxes indicate sections to be magnified for central regions of the defect, and the green dotted lines indicate the space occupied by bone graft particles. The red and blue boxes in (**d**) indicate the marginal and central regions of the defect, respectively, in the Col/Si-β-TCP@BMP-2 group to be magnified as H&E-stained images. Images of H&E-stained sections for (**e**) the marginal and (**f**) the central regions in the Col/Si-β-TCP@BMP-2 group. The yellow dotted lines indicate the marginal boundary of the bone defect. Abbreviations: NB, newly formed bone; LB, lamellar bone; OI, osteoid; CL, cutting line; V, vessel; OB, osteoblast; OC, osteocyte

Histological analyses were performed to evaluate the morphology of regenerated bone tissue. In gross images of MT-stained sections, a larger amount of bone tissue was formed newly within the defect of the Col/Si-β-TCP@BMP-2 group compared to the other groups (Fig. 4 a-d). The formation of new bones around the β-TCP particles (i.e., contact osteogenesis) was predominantly detected in the central region of defects of the β-TCP bone grafting groups, whereas the defect of the bare collagen plug group showed layers of primitive connective tissue without a detectable bone matrix synthesis. In the bone grafting groups, the bone graft particles melted out during the decalcification process and sites occupied initially by the particles are shown as empty spots. The Col/β-TCP and Col/β-TCP@BMP-2 groups exhibited clear boundaries between the β-TCP particle sites and surrounding bundles of collagen fibers. Notably, the Col/Si-β-TCP@BMP-2 group demonstrated a high degree of bone infiltration within blurred boundaries and maturely mineralized lamellar bone with the accumulation of osteoid adjacent to the Col/Si-β-TCP particles. From the images of H&E-stained sections, osteoblast rimming in the harversian canal and interspersed new vessels were observed in the Col/Si-β-TCP@BMP-2 group (Fig. 4e,f). These results indicate the faster transition of newly formed bone within the defect of the Col/Si-β-TCP@BMP-2 group into the bone maturation and remodeling phase.

## Discussion

The incorporation of growth factors has been considered as a promising approach for enhancing the biological performance of bone grafting substitutes [17, 27]. As a representative transforming growth factor-β (TGF-β) superfamily growth factor, BMP-2 regulates the growth, migration and differentiation of cells during bone formation and repair processes [28]. BMP-2 has been utilized for stimulating bone rehabilitation in clinical treatment of orthopaedic and dental bone defects [11, 12]. However, a retention of BMP-2 within the target site for a long period of time and in an effective and safe concentration is critical to achieve a maximal therapeutic performance; it is susceptible to be diluted and degraded in a physiological condition due to its short biological half-time [29], while excessive BMP-2 causes the compromised bone regeneration [30].

Given that composite approaches for incorporating several components often confer combined advantageous properties in terms of bioactivity and physicochemical properties, to accomplish successful therapeutic outcomes, we employed the composite approaches by incorporating the following three components. First, bioceramic β-TCP is recognized an effective carrier which has excellent biocompatibility and biodegradability, but the brittleness and insufficient plasticity are major hurdles for its extensive clinical applications [17, 27]. Second, collagen is a main component in ECM for structural organization as well as various interactions with cells [31]. Third, SiNPs-based vehicles have been considered as a controlled drug delivery system for keeping the drug concentration at a proper level for prolonged periods, improving therapeutic efficiency of the treatment [32]. In particular, the addition of biosilica not only improves bone regeneration [21] but also enhances the interfacial interactions within organic-inorganic composite materials [33]. Thus, we hypothesized the hybrid composites of collagen, β-TCP and biosilica can address drawbacks of each element and thus effectively enhance rehabilitation of bone defects.

As a proof-of-concept result, the Col/Si-β-TCP block showed ~ 16 % higher efficiency for BMP-2 entrapment than the non-silicated Col/β-TCP block, which can be coupled with strong interactions of the negatively charged biosilica with the positively charged BMP-2 [22]. In addition, this biosilicification-based composite approach led to 10-fold reinforcement of the compression strength (Fig. 1d), consistent with previous studies on the enhanced mechanical property by introducing silica [21, 34]. Notably, considering the compressive strength of conventional β-TCP block with 75 % porosity (~ 3 MPa) [35], mixing of both the Col/β-TCP and Col/Si-β-TCP blocks with collagen binder allows to toughen β-TCP block, similar to the reinforcement of long continuous fibers in toughening synthetic bone grafts such as calcium phosphate cements and sintered hydroxyapatite [36]. These results can be attributed to the long continuous fibers of collagen binder enables the β-TCP composite blocks to absorb higher amount of energy than sole β-TCP block by bridging the opening gap between the bone graft particles and dissipating the energy [37]. Collectively, we confirmed that biosilicated collagen and β-TCP composites can provide enhanced mechanical property to endure interfacial stress during *in vivo* service.

Even though the Col/Si-β-TCP@BMP-2 block exhibited the moderate effects on improving osteogenic differentiation *in vitro* for initial 2 weeks of incubation, the volume fraction of newly formed bone on the Col/Si-β-TCP@BMP-2 block was ~ 18 % higher than that on the Col/β-TCP@BMP-2 block in a calvarial bone defect *in vivo* (Fig. 3 c). We surmise that the improved interactions between the components in the Col/Si-β-TCP composite block enabled the robust retention of BMP-2 for initial 2 weeks and the significantly increase release after ~ 6 weeks post-implantation, eventually allowing the augmented bone regeneration of the Col/Si-β-TCP@BMP-2 block over the other control groups. Taken together, biosilicification of β-TCP bone grafts can be promising approach for robust BMP-2 delivery in a sustained manner to desired site of tissues.

## Conclusions

In the present study, we evaluated the Col/Si-β-TCP composites as an osteoinductive protein carrier and simultaneously an osteoconductive bone grafting material to accelerate bone regeneration. The simple adsorption of brown algae-derived E6Ectp1 peptide and subsequent biosilicification made it possible to incorporate and release the BMP-2 from the Col/β-TCP-based bone block in an active form and in a sustained manner, thus triggering the osteogenic behaviors and bone tissue formation. Even though more clinical trials and long-term follow-up studies are needed for further practical uses, we expect our BMP-2-delivering Col/Si-β-TCP composites can be as a promising alternative to autogenous graft.

## Data Availability

All data generated or analyzed in this study are included in this published article.

## References

[1] Nevins M, Jovanovic SA (1997). Localized bone reconstruction as an adjunct to dental implant placement. Curr Opin Periodontol.

[2] Bauer TW, Muschler GF (2000). Bone graft materials. An overview of the basic science. Clin Orthop Relat Res.

[3] Clavero J, Lundgren S (2003). Ramus or chin grafts for maxillary sinus inlay and local onlay augmentation: Comparison of donor site morbidity and complications. Clin Implant Dent Relat Res.

[4] Liu B, Lun DX (2012). Current application of beta-tricalcium phosphate composites in orthopaedics. Orthop Surg.

[5] Gao P, Zhang H, Liu Y, Fan B, Li X, Xiao X (2016). Beta-tricalcium phosphate granules improve osteogenesis in vitro and establish innovative osteoregenerators for bone tissue engineering in vivo. Sci Rep.

[6] Doi Y, Iwanaga H, Shibutani T, Moriwaki Y, Iwayama Y (1999). Osteoclastic responses to various calcium phosphates in cell cultures. J Biomed Mater Res.

[7] Baheiraei N, Nourani MR, Mortazavi SMJ, Movahedin M, Eyni H, Bagheri F (2017). Development of a bioactive porous collagen/β-tricalcium phosphate bone graft assisting rapid vascularization for bone tissue engineering applications. J Biomed Mater Res Part A.

[8] LeGeros RZ (2008). Calcium phosphate-based osteoinductive materials. Chem Rev.

[9] Shiwaku Y, Neff L, Nagano K, Takeyama K, de Bruijn J, Dard M (2015). The crosstalk between osteoclasts and osteoblasts is dependent upon the composition and structure of biphasic calcium phosphates. PLoS One.

[10] Laasri S, Taha M, Hlil EK, Laghzizil A, Hajjaji A (2012). Manufacturing and mechanical properties of calcium phosphate biomaterials. C R Mecanique.

[11] Triplett RG, Nevins M, Marx RE, Spagnoli DB, Oates TW, Moy PK (2009). Pivotal, randomized, parallel evaluation of recombinant human bone morphogenetic protein-2/absorbable collagen sponge and autogenous bone graft for maxillary sinus floor augmentation. J Oral Maxillofac Surg.

[12] Kim YJ, Lee JY, Kim JE, Park JC, Shin SW, Cho KS (2014). Ridge preservation using demineralized bone matrix gel with recombinant human bone morphogenetic protein-2 after tooth extraction: a randomized controlled clinical trial. J Oral Maxillofac Surg.

[13] Sohier J, Daculsi GU, Sourice S, de Groot K, Layrolle P (2010). Porous beta tricalcium phosphate scaffolds used as a BMP-2 delivery system for bone tissue engineering. J Biomed Mater Res Part A.

[14] Yang C, Unursaikhan O, Lee JS, Jung UW, Kim CS, Choi SH (2014). Osteoconductivity and biodegradation of synthetic bone substitutes with different tricalcium phosphate contents in rabbits. J Biomed Mater Res B Appl Biomater.

[15] Kakuta A, Tanaka T, Chazono M, Komaki H, Kitasato S, Inagaki N (2019). Effects of micro-porosity and local BMP-2 administration on bioresroption of β-TCP and new bone formation. Biomater Res.

[16] Kikuchi M, Ikoma T, Itoh S, Matsumoto HN, Koyama Y, Takakuda K, Shinomiya K, Tanaka J (2004). Biomimetic synthesis of bone-like nanocomposites using the self-organization mechanism of hydroxyapatite and collagen. Compos Sci Technol.

[17] Lu Y, Li M, Long Z, Yang D, Guo S, Li J (2019). Collagen/β-TCP composite as a bone-graft substitute for posterior spinal fusion in a rabbit model: a comparison study. Biomed Mater.

[18] Yang YC, Chang E (2001). Influence of residual stress on bonding strength and fracture of plasma-sprayed hydroxyapatite coatings on Ti-6Al-4V substrate. Biomaterials.

[19] Jo BH, Kim CS, Jo YK, Cheong H, Cha HJ (2016). Recent developments and applications of bioinspired silicification. Korean J Chem Eng.

[20] Yeo KB, Ki MR, Park KS, Pack SP (2017). Novel silica-forming peptides derived *from Ectocarpus siliculusus*. Process Biochem.

[21] Jo YK, Choi BH, Kim CS, Cha HJ (2017). Diatom-inspired silica nanostructure coatings with controllable microroughness using an engineered mussel protein glue to accelerate bone growth on titanium-based implants. Adv Mater.

[22] Macdonald ML, Samuel RE, Shah NJ, Padera R, Beben YM, Hammond PT (2011). Tissue integration of growth factor-eluting layer-by-layer polyelectrolyte multilayer coated implants. Biomaterials.

[23] Lee JH, Lee KM, Baek HR, Jang SJ, Ryu HS (2011). Combined effects of porous hydroxyapatite and demineralized bone matrix on bone induction: *in vitro* and *in vivo* study using a nude rat model. Biomed Mater.

[24] Niu LN, Jiao K, Qi YP, Nikonov S, Yiu CKY, Arola DD (2012). Intrafibrillar silicification of collagen scaffolds for sustained release of stem cell homing chemokine in hard tissue regeneration. FASEB J.

[25] Nefussi JR, Brami G, Modrowski D, Oboeuf M, Forest N (1997). Sequential expression of bone matrix proteins during rat calvaria osteoblast differentiation and bone nodule formation in vitro. J Histochem Cytochem.

[26] Huang W, Yang S, Shao J, Li YP (2007). Signaling and transcriptional regulation in osteoblast commitment and differentiation. Front Biosci.

[27] Zhu X, Zhang H, Zhang X, Ning C, Wang Y (2017). *In vitro* study on the osteogenesis enhancement effect of BMP-2 incorporated biomimetic apatite coating on titanium surfaces. Dent Mater J.

[28] Hallman M, Thor A (2018). Bone substitutes and growth factors as an alternative/complement to autogenous bone for grafting in implant dentistry. Periodontol 2000.

[29] Hunziker EB, Enggist L, Kuffer A, Buser D, Liu Y, Osseointegration (2012). The slow delivery of BMP-2 enhances osteoinductivity. Bone.

[30] Pelaez M, Susin C, Lee J, Fiorini T, Bisch FC, Dixon DR, McPherson JC, Buxton AN, Wikesjo UM (2014). Effect of rhBMP-2 dose on bone formation/maturation in a rat critical-size calvarial defect model. J Clin Periodontol.

[31] Roehlecke C, Witt M, Kasper M, Schulze E, Wolf C, Hofer A, Funk RW (2001). Synergistic effect of titanium alloy and collagen type I on cell adhesion, proliferation and differentiation of osteoblast-like cells. Cells Tissues Organs.

[32] Vallet-Regí M, Colilla M, Izquierdo-Barba I, Manzano M (2018). Mesoporous silica nanoparticles for drug delivery: Current insights. Materials.

[33] Rose S, Prevoteau A, Elzère P, Hourdet D, Maarcellan A, Leibler L (2014). Nanoparticle solutions as adhesives for gels and biological tissues. Nature.

[34] Shahroze RM, Ishak MR, Sapuan SM, Leman Z, Chandrasekar M, Asim M. Effect of silica aerogel additive on mechanical properties of the sugar palm fiber-reinforced polyester composites. Int. J. Polym. Sci. 2019;3978047.

[35] Kakuta A, Tanaka T, Chazono M, Komaki H, Kitasato S, Inagaki N, Akiyama S, Marumo K (2019). Effects of micro-porosity and local BMP-2 administration on bioresorption of β-TCP and new bone formation. Biomater Res.

[36] Ramay HRR, Zhang M (2004). Biphasic calcium phosphate nanocomposite porous scaffolds for load-bearing bone tissue engineering. Biomaterials.

[37] Krüger R, Groll J (2012). Fiber reinforced calcium phosphate cements - on the way to degradable load bearing bone. substitutes? Biomaterials.

